# The impact of temporal modulations in irradiance under light adapted conditions on the mouse suprachiasmatic nuclei (SCN)

**DOI:** 10.1038/s41598-017-11184-2

**Published:** 2017-09-05

**Authors:** Rachel Dobb, Franck Martial, Daniel Elijah, Riccardo Storchi, Timothy M. Brown, Robert J. Lucas

**Affiliations:** Faculty of Biology Medicine and Health, AV Hill Building, University of Manchester, Oxford Rd, Manchester M13 9PT UK

## Abstract

Electrophysiological responses of SCN neurons to light steps are well established, but responses to more natural modulations in irradiance have been much less studied. We address this deficit first by showing that variations in irradiance for human subjects are biased towards low temporal frequencies and small magnitudes. Using extracellular recordings we show that neurons in the mouse SCN are responsive to stimuli with these characteristics, tracking sinusoidal modulations in irradiance best at lower temporal frequencies and responding to abrupt changes in irradiance over a range of commonly encountered contrasts. The spectral sensitivity of these light adapted responses indicates that they are driven primarily by cones, but with melanopsin (and/or rods) contributing under more gradual changes. Higher frequency modulations in irradiance increased time averaged firing of SCN neurons (typically considered to encode background light intensity) modestly over that encountered during steady exposure, but did not have a detectable effect on the circadian phase resetting efficiency of light. Our findings highlight the SCN’s ability to encode naturalistic temporal modulations in irradiance, while revealing that the circadian system can effectively integrate such signals over time such that phase-resetting responses remain proportional to the mean light exposure.

## Introduction

Endogenous circadian clocks allow organisms to align their behaviour and physiology to 24 hour rhythms in the environment. In mammals, a master circadian oscillator is located in the hypothalamic suprachiasmatic nucleus (SCN) and is synchronised (entrained) to the external light-dark cycle via a direct projection from the retina (the retinohypothalamic tract; RHT). Discrete light pulses excite SCN neurons and reset circadian phase according to stimulus irradiance and duration, and the circadian time at which it is presented^1^. The RHT is dominated by a particular class of retinal ganglion cell (M1 type ipRGCs) that express melanopsin and are directly photosensitive^2–6^. ipRGCs also receive synaptic input from the outer retina^7, 8^ and as a consequence, their light response is a composite of intrinsic (melanopsin) and extrinsic (rod and cone) influences.

The inclusion of rods and cones in the RHT enhances its sensitivity, allowing the clock to respond to stimuli below the threshold for melanopsin photoreception^9, 10^. However, it also has other consequences for the RHT’s sensory capacity, including rendering it sensitive to colour and spatial patterns^11, 12^. Here we consider the case of temporal modulations in irradiance. Both rods and cones are especially responsive to abrupt increases in light intensity and this characteristic is apparent in the light response of the RHT. Thus, when presented with a light pulse from darkness, both ipRGCs and visually responsive SCN neurons exhibit a transient hyper-excitation at lights on that relaxes to a lower steady state activity under continuous illumination^2, 13–17^. An important unanswered question is the extent to which this phenomenon also impacts the SCN response to more commonly encountered variations in irradiance under light adapted conditions. Are SCN neurons similarly over-excited by abrupt increases in irradiance when presented against a background light? If so, what magnitudes and rates of change most effectively elicit such effects? What impact do such phenomena have on the ability of the SCN to encode long-term average irradiance (which provides the most reliable indication of time-of-day)? Finally, what are their consequences, if any, for the circadian phase resetting efficiency of light?

## Results

### Natural variations in irradiance

To provide a frame of reference for our exploration of the SCN’s response to temporal variations in irradiance we first set out to define typically encountered patterns of irradiance exposure in humans. To this end, we fitted a photodiode detector sampling irradiance at 5 Hz to a spectacles frame oriented so that the detector surface was parallel to the subject’s cornea. This recorded variations in irradiance caused by changes in the local environment, or head and body (but not eye) movements of the subject. The detector did not track variations in spectral composition of light, but had a spectral sensitivity function close to V(λ) allowing us to calibrate it to record illuminance (units = lux; methods).

**Figure 1:**
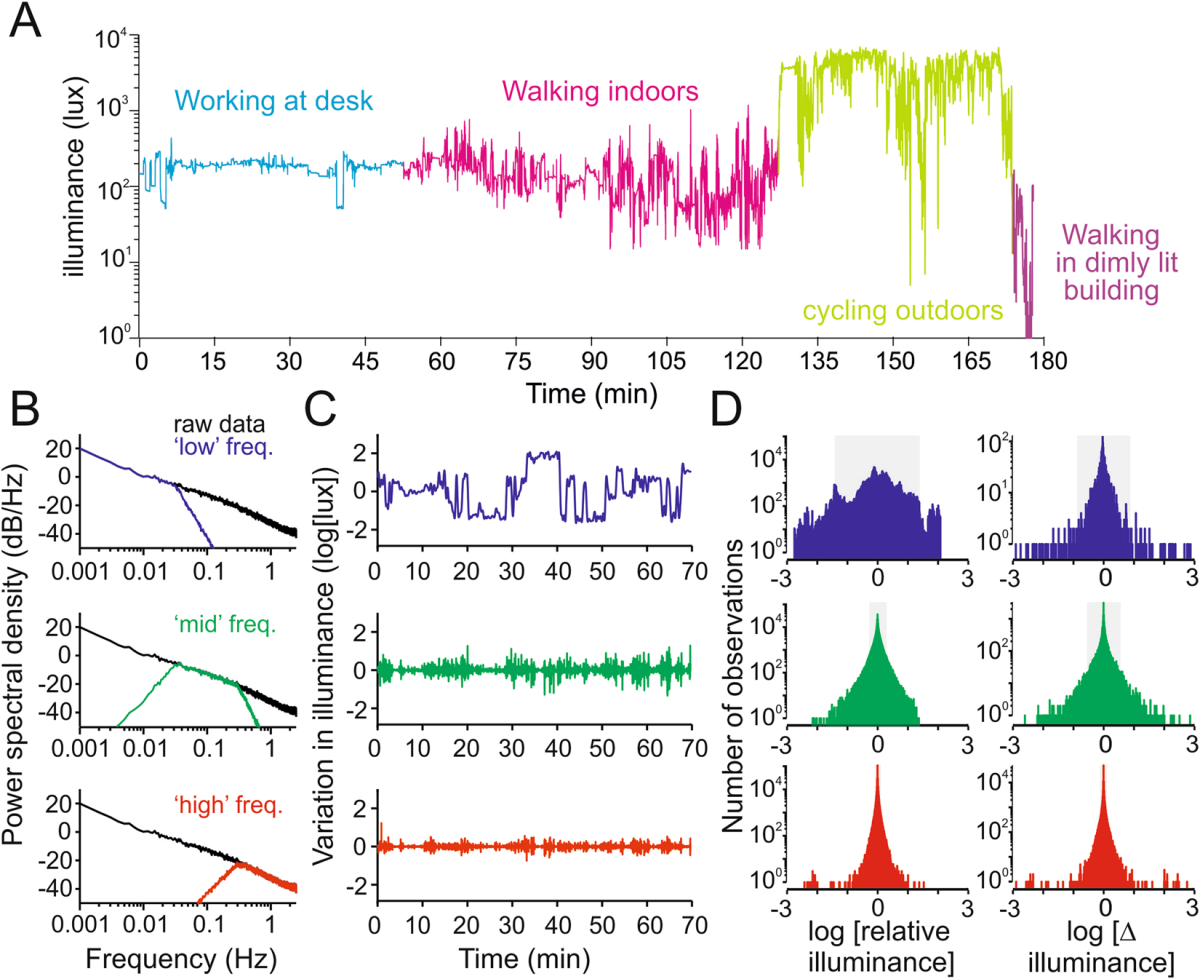
Natural variations in illuminance. (**A**) Example of a raw field recording encompassing a variety of activities and lighting environments. (**B**) Power spectral densities for natural variations in illuminance derived from the full dataset (a weighted average of 11 traces each comprising 0.6–3 h of data across a wide range of lighting conditions and activities, total = 14.34 h). Traces show relationship between power and temporal frequency for the raw data and after the filtering operations used to extract ‘low’, ‘mid’ or ‘high’ frequency components of the signal for subsequent analysis. (**C**) Sample segment of illuminance recording (walking between indoor and outdoor environments) filtered to extract ‘low’, ‘mid’ or ‘high’ frequency components. Note: low frequency component was normalised by subtracting the mean illuminance across the whole segment (in this case 2.84 log Lux). (**C**) Histograms showing distribution of illuminance values (relative to mean for each recording epoch; left) or moment to moment changes in illuminance (delta between local minima and maxima) across the full dataset and within the frequency bands indicated in (**A**). Shaded regions represent the central 95% of each data distribution.

A typical record from a person wearing this detector undertaking a range of activities is shown in Fig. 1A. As expected, illuminance was markedly lower when indoors, while its variability was highest during epochs of physical activity. We subjected a larger dataset comprising 11 recordings (total recording time 14.34 hrs) collected outdoors while sitting, walking, cycling or driving in Manchester (UK) or Milan (Italy), or indoors predominantly while working at a desk, to a power spectrum analysis to assess the timescales over which illuminance varied. This revealed a strong negative correlation between power and temporal frequency, indicating that most of the variation in illuminance occurred over longer timescales (Fig. 1B). To explore the magnitude of variations in illuminance typically encountered during the recordings, we split the dataset in ‘low’, ‘mid’ and ‘high’ frequency components. A typical 70 min recording epoch filtered in this way is shown in Fig. 1C. A comparison of the distribution of illuminance values, or the difference in illuminance between local minima and maxima, across the whole dataset confirmed that larger variations in illuminance occur at lower temporal frequencies (Fig. 1D). Moreover, it was rare to encounter variations in illuminance as high as 10x over any frequency range (Fig. 1D). These fundamental properties were retained when the data were separated into outdoor and indoor epochs (Supplementary Fig. 1).

### SCN responses to light steps

We started our exploration of SCN responses to variations in irradiance under light adapted conditions by presenting steps at a range of contrasts. We presented 1 s steps in irradiance whose magnitude (x2 to × 24 fold) covered the range encountered in our environmental recordings, against a range of background irradiances (10^14^, 10^13^ and 10^12^ photons/cm^2^/s) while recording extracellular activity in the SCN of anaesthetised mice. We used C57Bl6 mice with a fully intact visual system, but in which a coding sequence for the human red (LWS) cone opsin had been knocked into the mouse MWS cone opsin locus^18^. In these animals, cone photosensitivity is shifted towards longer wavelengths and away from that of melanopsin, allowing changes in the spectral composition of visual stimuli to be used to identify the separate contribution of cone vs melanopsin photoreception to evoked responses^16, 19^.

**Figure 2:**
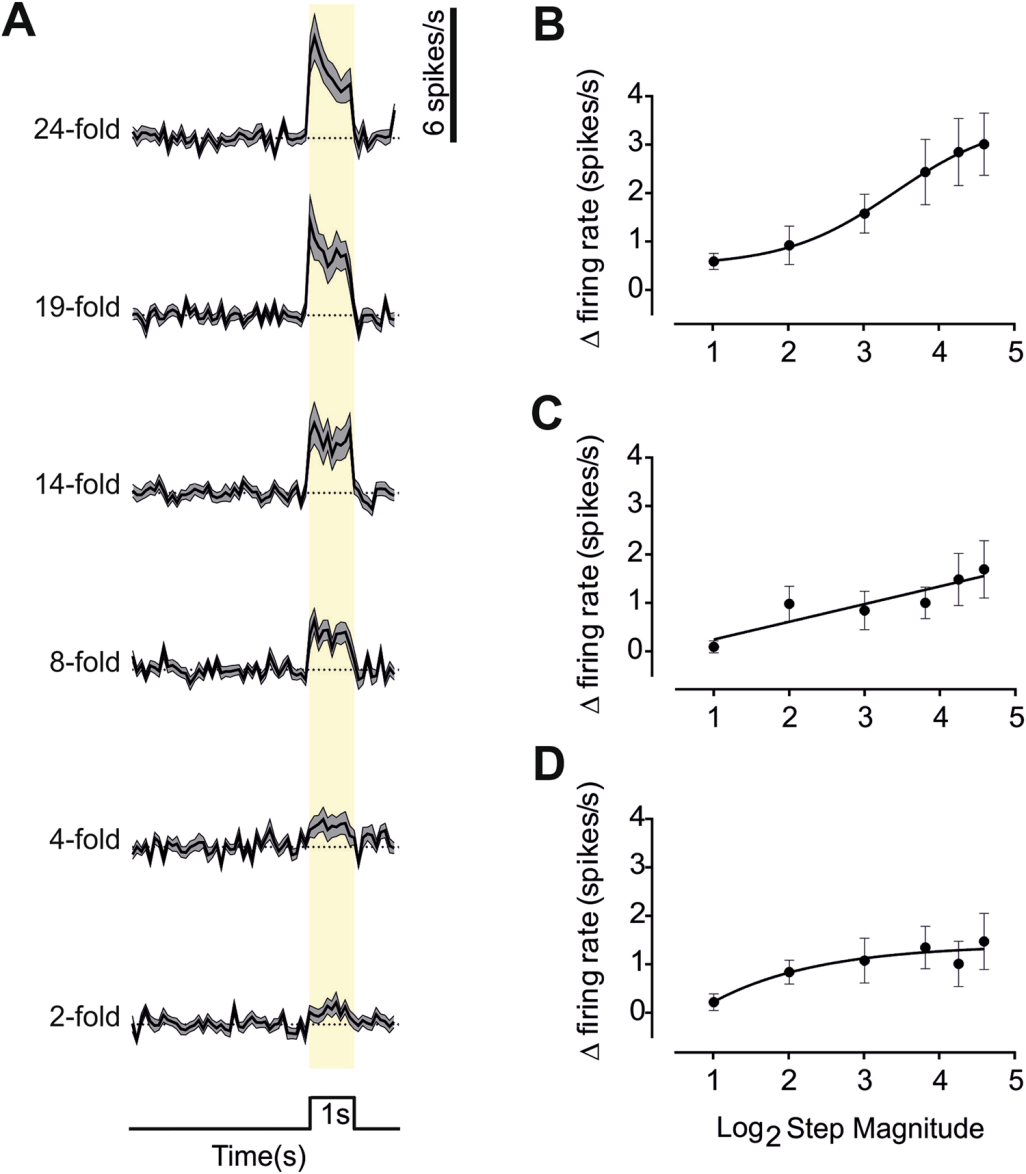
SCN responses to steps at a range of contrasts and background light intensities. (**A**) Changes in firing rate (mean ± SEM baseline subtracted; scale to right) in response to 1 s light steps (timing indicated below and with yellow shading; 5 s inter-step interval) over a range of magnitudes (fold change in irradiance indicated to left) at the brightest background (10^14^ photons/cm^2^/s) for 46 light responses single units from 19 mice. (**B**,**C** and **D**) The relationship between response amplitude (Mean ± SEM increase in firing during the 1 s light step) and increment magnitude (fold change) at the three different backgrounds, ‘Bright’ (**B**, n = 46, 19 mice), ‘Moderate’ (**C**, n = 39, 9 mice) and ‘Dim’ (**D**, n = 31, 9 mice). Each is fitted with a sigmoidal curve.

Aggelopoulos and Meissl^14^ have previously reported that units in the rat SCN predominantly respond to light steps under photopic conditions with an ON excitation followed by OFF inhibition. In our recordings, 46 out of 98 single units from 19 mice showed a significant response to the highest contrast step. In each case, the most reliable element of the response was ON excitation (Fig. 2A), with OFF inhibition, if present, less prominent. The ON response itself comprised an initial abrupt increase in firing, that relaxed to a lower level activity sustained throughout the 1 s step (Fig. 2A; mean ± SEM firing rate (spikes/s) across units at background = 4.147 ± 0.858, peak = 11.435 ± 1.603 and across the 1 s step = 7.159 ± 1.294).

The response to the × 24 step was recapitulated for smaller increases in irradiance (Fig. 2B), although response magnitude was strongly dependent upon step amplitude (RM one-way ANOVA p < 0.0001, Fig. 1C). Qualitatively similar responses were recorded for steps of equivalent relative magnitude presented against backgrounds of 10x or 100x lower irradiance (‘moderate’ or ‘dim’ conditions; Fig. 2C and D). Peak response amplitude (for x 24 step) was however significantly impacted by ambient irradiance (mean ± SEM increase in firing rate (spikes/s) 3.01 ± 0.64, 1.69 ± 0.59, 1.47 ± 0.58 for bright, medium and dim backgrounds respectively, p < 0.05 Kruskal-Wallis 1-way ANOVA).

We next asked which photoreceptors contribute to the SCN’s ability to track dynamic changes in light intensity. One might imagine that cones play a dominant role, as their ability to encode changes in luminance under light adapted conditions is fundemental to their role in pattern vision. To test this we recorded responses to a simple temporal modulation in irradiance (1 s steps at 5 s ISI) visible only to cones. We achieved this using the principles of receptor silent substitution to generate changes in spectral composition that amounted to an increase in effective irradiance for UVS and MWS cone opsins but not for rods or melanopsin (see methods).

**Figure 3:**
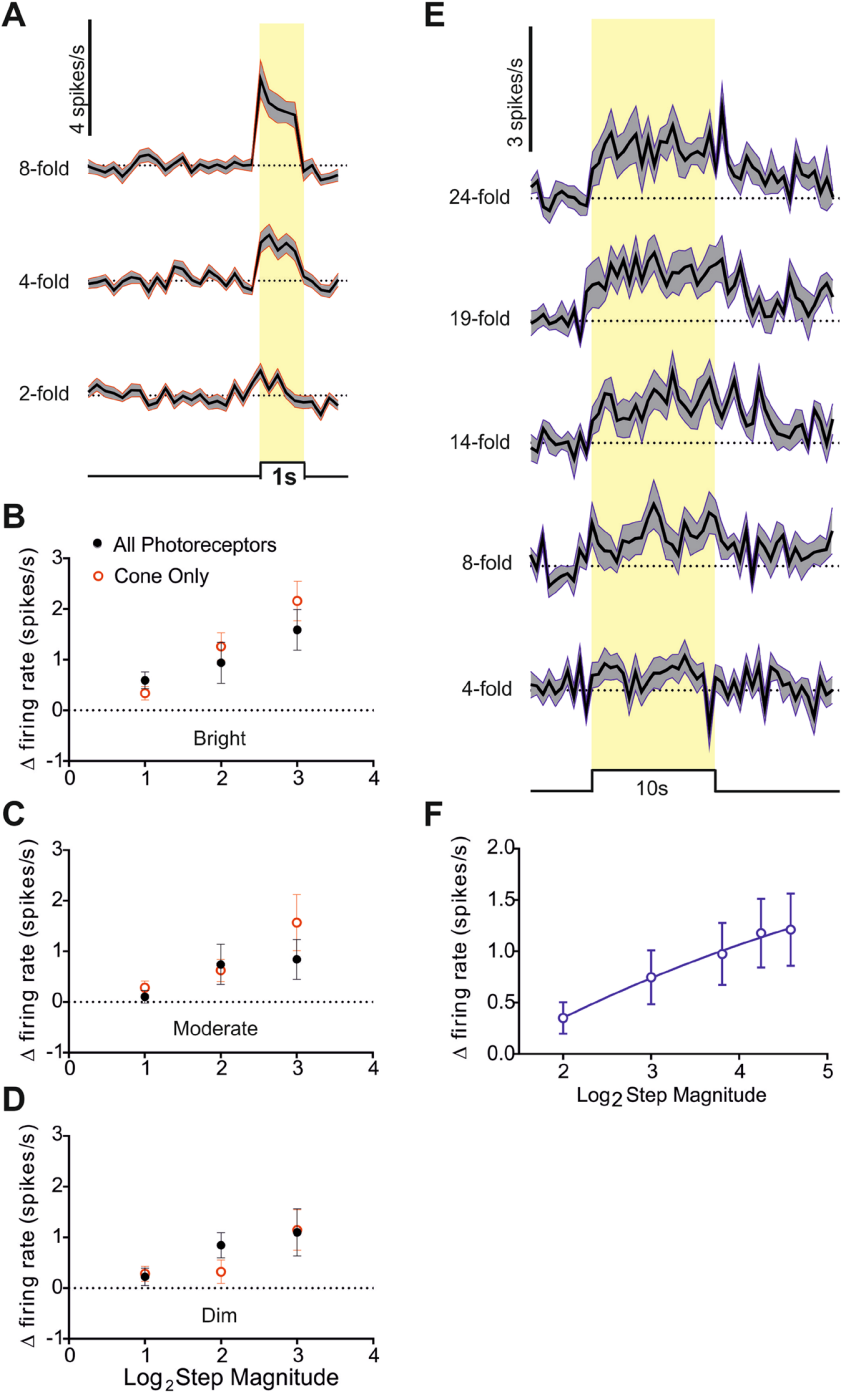
Cone and melanopsin contributions to step responses. (**A**) Changes in firing rate (mean ± SEM baseline subtracted; scale at top left) in response to 1 s light steps (timing indicated below and with yellow shading; 5 s inter-step interval) visible only to cones over a range of magnitudes (fold change in irradiance indicated to left) for 46 light responses single units from 19 mice at the brightest background (10^14^ photons/cm^2^/s). (**B**,**C** and **D**) Comparisons of response amplitude (mean ± SEM change in firing) for 1 s stimuli presenting × 2, × 4 and × 8 increases in effective irradiance visible to all photoreceptors (black) or cones alone (red) at bright (**B**) 10^14^ photons/cm^2^/s, n = 46 units, 19 mice), moderate (**C)** 10^13^ photons/cm^2^/s, n = 39, 9 mice) or dim (**D**) 10^12^ photons/cm^2^/s, n = 31, 9 mice) backgrounds. (**E**) Changes in firing rate (mean ± SEM baseline subtracted; scale at top left) in response to 10 s light steps (timing indicated below and with yellow shading; 5 s inter-step interval) visible only to rods and melanopsin over a range of magnitudes (fold change in irradiance indicated to left) for 14 light responses single units from 10 mice at the brightest background (10^14^ photons/cm^2^/s). (**F)** Quantification of response amplitude (mean ± SEM increase in firing across 10 s step) as a function fold change in effective irradiance for ‘melanopsin only’ steps. Data are fit with a sigmoidal curve.

SCN neurons showed qualitatively similar responses to these ‘cone’ stimuli (Fig. 3A) as they had to simple light steps (termed ‘all photoreceptor’ stimuli; Fig. 2A) matched for cone contrast but providing increases in irradiance visible also to rods and melanopsin. To facilitate quantitative comparisons we interleaved the ‘cone’ stimuli at increases in effective irradiance of x2, x4 and x8 with equivalent ‘all photoreceptors’ steps. We found that the change in firing rate was equivalent between ‘cone’ and ‘all photoreceptors’ across this range of contrasts (Fig. 3B; p = 0.471 for x2, p = 0.098 for x4 and p = 0.14 for x8; Mann Whitney two-tailed tests). This similarity between cone and all photoreceptors responses was retained for the moderate and dim backgrounds (Fig. 3C and D). These data indicate that under these conditions, and at least for the relatively modest changes in irradiance that were most common in our field recordings, SCN responses to abrupt and fairly transient modulations in irradiance (1 s steps) can be adequately explained by the activity of cone photoreceptors alone.

The SCN response to light pulses under dark-adapted conditions is thought to recruit melanopsin for sustained changes in irradiance^11, 16, 20, 21^. To determine the contribution of melanopsin and/or rods to more dynamic changes in irradiance we therefore turned to longer steps in irradiance and employed the silent substitution approach to produce steps (up to 24x increase) in effective irradiance visible to melanopsin and rod, but not cone, photoreceptors (methods). Since we applied these stimuli (10 s steps; 130 s inter-stimulus interval) at the brightest background (10^14^ photons/cm^2^/s) they should strongly favour melanopsin over rod activity. Such ‘melanopsin’ stimuli induced significant increases in firing (two tailed t-test p < 0.05 for firing rate during last 5 s of step compared to 10 s baseline before stimulus) in 14 out of 25 light responsive SCN units from 10 mice (Fig. 3E). Response amplitude was positively correlated with the fold change in rod/melanopsin effective irradiance (Fig. 3F; RM one-way ANOVA p = 0.0022). For the largest magnitude (x24) step the mean ± SEM change in firing was 1.2 ± 0.3 spikes/s, and for all stimuli, the temporal profile of the response (Fig. 3E) was similar to that previously reported for melanopsin driven activity^22^, building up slowly after lights on and dissipating gradually after lights off.

### Responses to graded changes in irradiance

**Figure 4:**
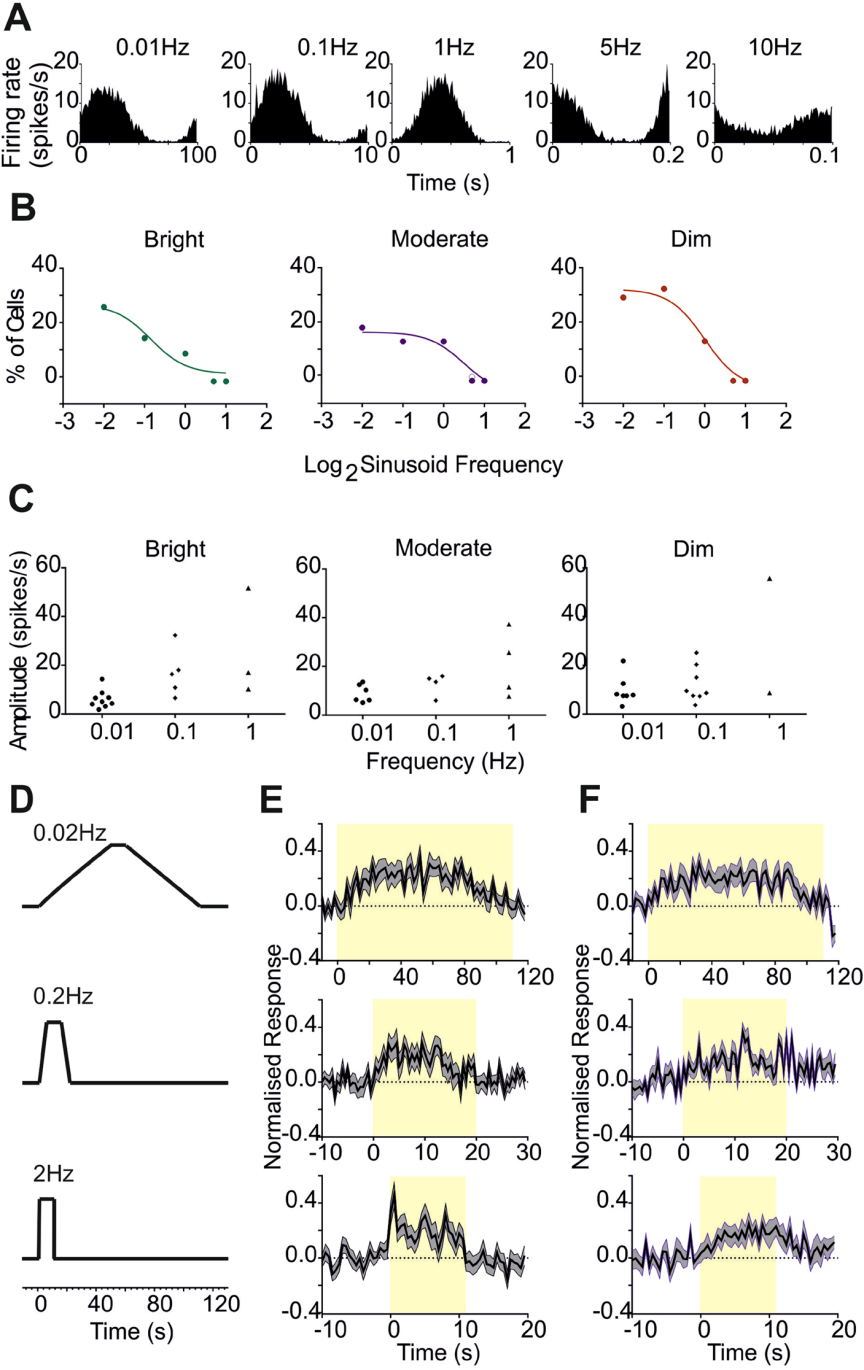
Responses to continuous modulations in irradiance. **(A**) Modulations in firing rate for an example cell with unusually broad temporal frequency sensitivity in response to sinusoidal modulations (24x trough:peak increase in irradiance) presented at 0.01, 0.1, 1, 5 and 10 Hz at the brightest background (10^14^ photons/cm^2^/s). (**B**) Percentage of light responsive units showing significant tracking of the sinusoids (see methods) at each frequency at bright (10^14^ photons/cm^2^/s; left), moderate (10^13^ photons/cm^2^/s, middle) and dim (10^12^ photons/cm^2^/s, right) backgrounds. (**C)** Peak to trough amplitude of modulation in firing rate (spikes/s) for each unit with a significant response to sinusoids at 0.01 – 1 Hz at each background. (**D)** Schematic of light presentation profiles for gradual (top: ramp up time = 50 s), moderate (middle: ramp up time = 5 s), and fast (bottom: ramp up time = 0.5 s) ramps (x24 peak:trough increase in irradiance). (**E**) Normalised response (mean ± SEM change in firing rate baseline subtracted and normalised for 1 = maximum firing for that unit under any condition) in response to ramp stimuli in (**D**) visible to all photoreceptors (n = 25 light responsive units). (**F**) Normalised response (mean ± SEM change in firing rate baseline subtracted and normalised for 1 = maximum firing for that unit under any condition) in response to ramp stimuli in (**D**) rendered visible only to rods and melanopsin (n = 14 units responsive to ‘melanopsin’ steps).

We next turned our attention to the rates of change in irradiance to which the SCN is responsive, initially by exploring its temporal frequency tuning. Given the preponderance of low frequency elements in our field recordings, we presented sinusoidal modulations in irradiance (‘All photoreceptors’ stimuli; x24 peak to trough amplitude) over a range of low to moderate frequencies (0.01, 0.1, 1, 5, 10 Hz). We found examples of neurons whose firing rates appeared strongly modulated at all of these frequencies (Fig. 4A shows the activity of one such unit), however responses were most common at the lower frequencies. Thus, a power spectrum analysis returned up to 30% of the light responsive neurons as tracking 0.01 or 0.1 Hz stimuli (power at stimulus frequency >2.5 standard deviations above baseline) at each of 3 backgrounds tested (‘bright’, ‘moderate’ or ‘dim’; Fig. 4B). Only ~10% of cells tracked the 1 Hz oscillation (although often with high amplitude; Fig. 4C), and responses to the two highest frequencies were found in only one or two units at each background. These data reveal that the SCN most actively tracks lower frequencies, matching the appearance of such events in our field recordings.

We next set out to define the relative contribution of cones and melanopsin to tracking gradual changes in irradiance. For this purpose we turned to ramp stimuli in which we could separately record responses to irradiance increments and decrements. To this end, we presented irradiance ramps at 3 rates of change (24 fold increase in irradiance over 0.5, 5 or 50 s; Fig. 4E) and, using the silent substitution method, visible either to all photoreceptors (‘all photoreceptors’ stimuli) or only to rods and melanopsin (‘rods/melanopsin’). In each case, irradiance ramped from baseline to maximum over the defined period, was held at that level for 5 s before ramping down to baseline at the same rate. ‘All photoreceptors’ ramps at all 3 rates of change modulated the firing rate of many SCN neurons (Fig. 4F; n = 25, mean ± SEM amplitude of modulation = 0.701 ± 0.379 spikes/s for the slowest ramps, 1.317 ± 0.786 spikes/s for the intermediate rate of change, and 1.799 ± 0.968 spikes/s for the fastest ramp). The normalised mean response across units appeared asymmetrical for even the slowest ramp. However, only the fastest ramp induced the characteristic ‘On’ hyper-excitation elicited by 1 s pulses (Fig. 4F).

Responses to the ‘melanopsin’ ramps were hard to discern from the total population. We therefore first filtered the dataset for those units with statistically significant responses to the simple 10 s ‘melanopsin’ step (n = 14). This subset of cells showed a modulation in firing rate to all 3 ‘melanopsin’ ramps (Fig. 4G). In common with their response to the 10 s stimulus (Fig. 3E), there was no abrupt ‘On’ transient response for even the fastest ‘melanopsin’ ramp. At later timepoints in the stimulus, activity recorded under ‘melanopsin’ and ‘all photoreceptors’ stimuli converged. These data thus indicate that while melanopsin can be responsible for part of the modulation in SCN firing produced by these ramps, cones also make a significant contribution even for the most gradual change in irradiance trialed here.

### Impact on time averaged firing

**Figure 5:**
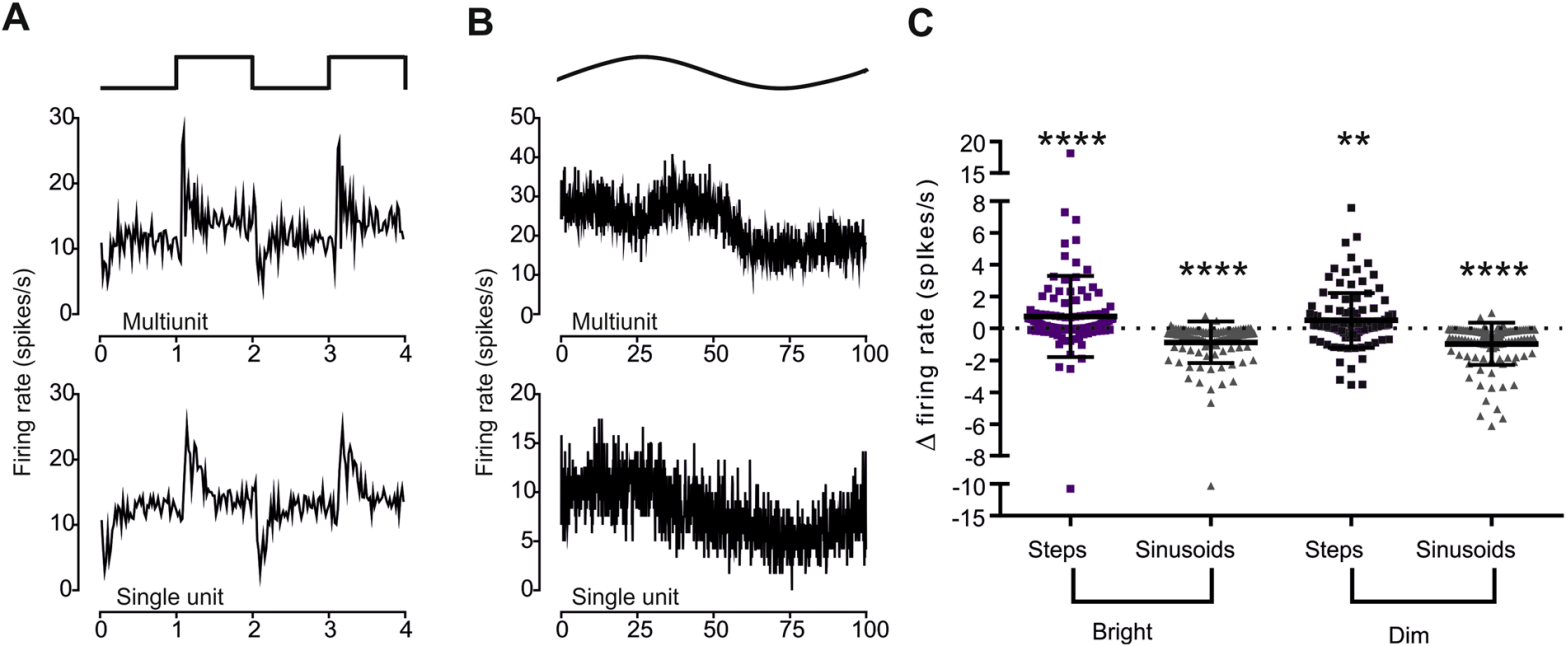
The impact of temporal modulations in irradiance on time averaged firing rate. (**A)** Example multiunit (top) and single unit (bottom) traces of firing rate (mean spikes/s over 120 repeats) over 2 cycles of 0.5 Hz step stimulus (profile above). (**B)** Example multiunit (top) and single unit (bottom) traces of firing rate (mean spikes/s over 3 repeats) over 2 cycles of 0.01 Hz sinusoid stimulus (profile aove). (**C**) Change in multiunit activity (spikes/s) at 112 recording sites (from 10 mice) between baseline (steady light) and step (squares) or sinusoidal (triangles) modulations in irradiance (as in **A** and **B**) at bright (10^14^ photons/cm^2^/s) and dim (10^12^ photons/cm^2^/s) backgrounds. Annotated to show mean ± SEM. Activity was higher under steps, and lower under sinusoids, at both backgrounds (Wilcoxon Signed Rank test; **P < 0.01; ****P < 0.0001).

Both ipRGCs and SCN neurons encode the irradiance of extended light steps in their time averaged firing rate^2, 8, 13, 16, 17^. The response of SCN neurons to changes in irradiance described above reveals that at a finer timescale SCN firing is also impacted by the direction and rate of change in irradiance. We next asked whether this latter influence on SCN firing impaired its ability to encode long-term average irradiance. To this end, we recorded activity under sequential 5 min epochs under either a steady light or an equivalent light dose with temporal modulation (24x peak: trough difference in irradiance) presented as 1 s steps (0.5 Hz) 5or a 0.01 Hz sinusoidal oscillations. Consistent with the other findings presented here, SCN firing rates in single and multi-unit activity tracked both sinusoids and steps (Fig. 5A and B). For comparisons of time averaged firing we employed multi-unit activity as a representation of global SCN activity. Time averaged firing rates were modestly, but statistically significantly, enhanced by the inclusion of ‘steps’ compared to the steady light (Fig. 5C; mean ± SEM difference in firing rate between steady light and steps 0.753 ± 0.24 and 0.514 ± 0.16 spikes/s for ‘Bright’ and ‘Dim’ conditions respectively, Wilcoxon Signed rank test p < 0.01 for both; firing rates under steady light 6.425 ± 0.79 and 7.041 ± 0.86 spikes/s). Conversely, firing rate under the slow sinusoids was significantly reduced (Fig. 5C; Wilcoxon Signed rank test p < 0.0001 for both ‘Bright’ and ‘Dim’ backgrounds, mean ± SEM difference in firing rate for the ‘Bright’ condition is −0.863 ± 0.12 spikes/s, compared to −0.956 ± 0.12 for the ‘Dim’).

### Circadian phase resetting impact of temporal modulations

**Figure 6:**
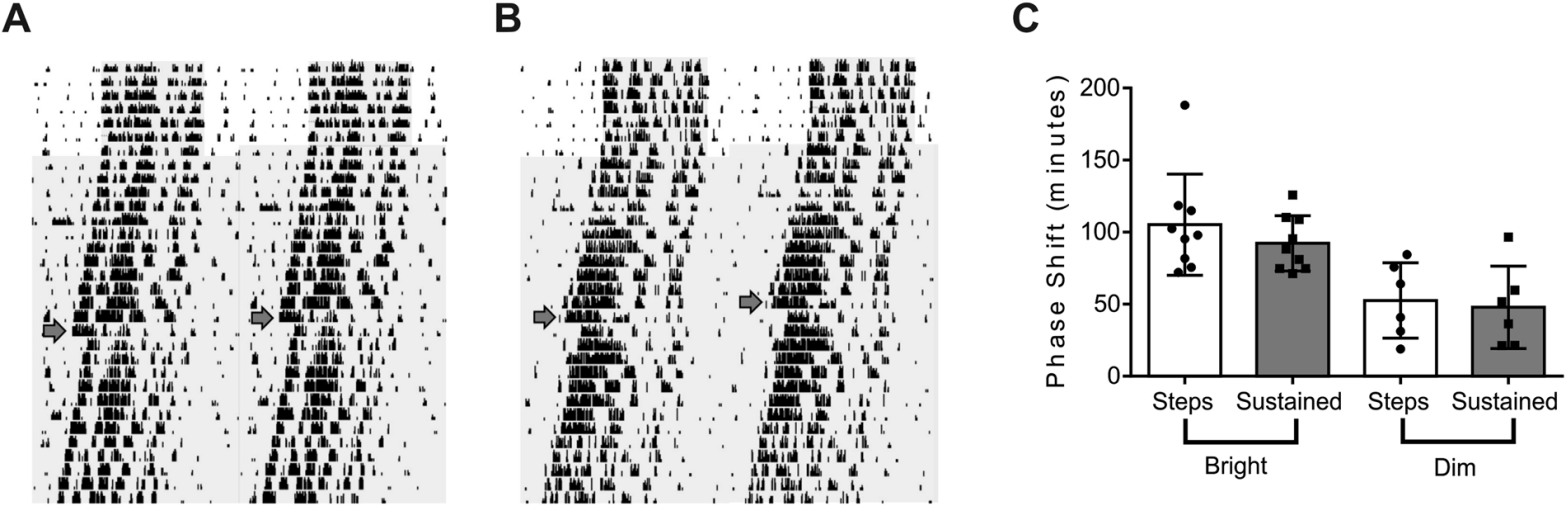
Phase shifts in circadian rhythm of locomotor activity. (**A** and **B**) Example double plotted actograms for mice initially entrained to 12:12LD cycle (first 5 days) and then free-running in constant darkness and exposed to a 15 min light pulse at CT16 comprising either sustained exposure (**A**) or 0.05 Hz steps (**B**) matched for total photon flux on day indicated by arrow. Note shifts in activity rhythm associated with light exposure in both animals. (**C**) Phase shift amplitude (mean ± SEM) for mice associated with 15 min light pulses in the form of steps 0.05 Hz steps (‘Steps’; green) or steady exposure (‘sustained’, grey) matched to provide 14.5 log photons/cm^2^/s (‘Bright’) or 12.2 log photons/cm^2^/s (‘Dim’).

We finally set out to determine the impact of temporal modulations in irradiance on one important function of the RHT, circadian phase resetting. Locomotor activity rhythms were recorded in mice free running in constant darkness and exposed to a 15 min light pulse at circadian time 16 (4 hours after habitual activity onset; Fig. 6A). Responses to versions of this stimulus matched for total photon flux (either 14.5 or 12.2 log photons/cm^2^/s) but either invariant or comprised of 1 s pulses at 1 Hz were compared. As predicted all stimuli induced clear shifts in the circadian rhythm, and shifts were significantly larger for the higher irradiance for both steady and modulated light (Fig. 6B; p = 0.008 and 0.003 respectively, unpaired t-test). There was no significant impact of the temporal pattern however, with equivalent responses to steady and modulated light at each irradiance (p = 0.45 and p = 0.78 for high and low irradiance respectively, Mann Whitney test).

## Discussion

Laboratory studies of circadian photosensitivity have traditionally relied upon analytical light exposure paradigms comprising extended exposure to stimuli of invariant intensity. These have the advantage of being easy to control and quantify and have been extremely useful in establishing the fundamental characteristics of the circadian visual system. It seems obvious, however, that real world patterns of light exposure would be more complex. Our measures of corneal irradiance recorded while undertaking common activities confirm that this is indeed the case, with variations in irradiance occurring over a wide range of frequencies. Abrupt changes in irradiance occur under all circumstances (likely associated with changes in direction of view), but we also see strong correlations over time.

It follows, that under natural conditions the retinal ganglion cells innervating the SCN are exposed to modulations in irradiance over a very wide range of timescales from months to milliseconds. The lower frequencies may contain information about time of day, month and year, while more abrupt changes in irradiance do not. This explains the preponderance in published SCN studies of extended light exposures. In terms of the best-understood function of the RHT (circadian entrainment), higher frequency modulations represent noise and could easily be excluded by including a low pass temporal frequency filter at some point in the circadian photoentrainment pathway. Indeed, we show here that inclusion of temporal contrast does not change the phase resetting efficiency of light. However, we also show that modulations over a range of contrasts and frequencies are faithfully reflected in the firing pattern of SCN neurons.

If one accepts the argument that spike firing of SCN neurons is mechanistically related to circadian phase shifts, then our data beg the question of why temporal modulations in irradiance that impact SCN firing do not alter circadian resetting. One simple answer is that their major impact is on the fine timing of spikes rather than on time averaged activity. We find here that temporal modulations have only a small effect on that latter parameter. A circadian phase shifting mechanism dependent upon integrated firing over many seconds would therefore be relatively isolated from the effects of higher frequency modulations in firing and predominantly influenced by long-term variations in the intensity and/or spectral composition of ambient light. The circadian system does indeed appear to have such long-term temporal integration properties^23^.

It is interesting, however, to consider instances in which temporal modulations have been reported to influence circadian phase shifts. One instance is for transgenic mice in which stimuli strongly targeting cone photoreceptors can be applied^9, 21^. As we see here that cones are disproportionately responsible for the SCN’s ability to track fast changes in irradiance, the impact of temporal modulations on time averaged firing (and the clock) might be expected to be greater in conditions in which rod and melanopsin activity was relatively low. The other documented instance of temporal modulations altering circadian photosensitivity is for very brief light pulses^24–26^. Such stimuli minimize light adaptation in all photoreceptor classes and thus hyper-excite the RHT and circadian clock. That interpretation is consistent with the ability of flickering light to increase the firing rate of ipRGCs, the pupil light reflex^15, 27^ and, indeed perceived brightness (Brucke-Bartley effect). The data presented here argue however that while such very brief light stimuli may represent the opportunity to produce what might be called ‘circadian visual illusions’ in which the clock responds as if exposed to more light than it actually was, the circadian resetting mechanism is likely buffered against the impact of more commonly encountered variations in irradiance.

An important practical question in circadian biology is how best to estimate the circadian phase resetting efficiency of a particular light exposure regime. Our data address the likely impact of two dimensions in which light exposure can vary – its spectral composition, and its temporal profile. In terms of the latter, we find that while the SCN is well capable of encoding naturally encountered temporal modulations in irradiance, these likely have little impact on circadian photosensitivity and thus that a time averaged record of irradiance likely provides an adequate method of summarizing light exposure for the clock. The issue of spectral composition is more complex. Our data do not address the role of colour in circadian entrainment^11^ but rather the impact of wavelength composition on effective irradiance. Because biological photoreceptors are differentially sensitive to light across the spectrum, any attempt to characterize intensity for a polychromatic light source has to apply a weighting function matching that of the photoreceptor(s) subserving the biological response of interest. In this context, it is important to predict whether cones or melanopsin play the dominant role in encoding irradiance under natural field conditions^28^. Our data indicate that, over the range of light intensities that typify the night-day transition, cones provide the dominant mechanism for driving SCN responses to the coincident moderate-high frequency modulations in irradiance experienced in the natural world. Cones are thus likely to be a substantial influence on the pattern of firing in the SCN under natural daytime conditions. On the other hand, our data also show how such dynamic modulations in firing can exist without substantially altering either the circadian phase resetting efficiency of light or the impact of more sustained variations in irradiance on maintained firing rate. The relative importance of cones and melanopsin to tracking diurnal variations in irradiance thus remains to be determined.

If the responses to temporal modulations in firing recorded here do not impact circadian phase resetting, could they have some other functional significance? Future work will be required to address this question. It may be that they are a passive reflection of cone input to the RHT and have no function. However, the SCN coordinate light dependent changes in behaviour and physiology quite independent of their circadian rhythm generation capacity. Thus far, such responses have been explored using long lasting changes in irradiance. However, there is evidence that other visual features can be relevant for the SCN^29^. The temporal pattern of corneal irradiance could be a rich source of information about our local environment and the behaviour of objects within it useful, for example, for identifying situations of heightened threat such as stepping out from shade. The SCN’s connections to influential autonomic and neuroendocrine systems provide an efficient route via which to induce appropriate changes in behavioural and physiological state.

## Methods

The datasets generated during the current study are available from the corresponding author on reasonable request.

### Animals

Mice were bred at the University of Manchester and housed under a 12:12 light/dark cycle, with food and water available ad libitum. Male adult red cone mice, C57Bl6 *Opn1mw* ^*R*^ ^9, 18^ were used in all electrophysiological experiments, aged 3–6 months (80–160 days). All procedures were approved by the University of Manchester Animal Welfare Ethical Review Board or University Research Ethics Committee and conducted according to the United Kingdom Animals (Scientific Procedures) act, 1986.

### *In vivo* neurophysiology

Mice were anaesthetised with an intraperitoneal (i.p.) injection of urethane (1.6 g/kg; 30% or 20% w/v; Sigma-Aldrich, Poole, UK), with additional i.p. or subcutaneous injections of urethane (0.2 gkg^−1^) as necessary to maintain anaesthesia and held in a stereotaxic apparatus (SR-15M; Narishage International Ltd, London, UK) with homoeothermic control (heat mat Harvard Apparatus, Edenbridge, UK). After exposing the skull surface, a small hole was drilled above the SCN (0.4mm anterior to bregma, 0.1mm lateral to the midline and between 5.5 and 6.5mm ventral to the cortical surface). Atropine (Sigma-Aldrich) and Mineral oil (Sigma-Aldrich) were applied to eyes, the former to achieve pupil dilation and the latter to retain corneal moisture.

Linear silicon arrays consisting of 32 channels were used for SCN recordings. Recording probes (Neuronxus Technologies Inc., Ann Arbor, MI, USA) either consisted of a single shank (A1 x32–10mm-50–413 or A1 x32Poly) or four shanks (Buszaki32L). Recording probes were centred on the craniotomy and then lowered into the brain (to a depth of between 5.3 and 6.3mm relative to the brain surface) so that the recording sites spanned the SCN, using a fluid filled micromanipulator (MO-10; Narishige). 2 s flashes of bright light were used to confirm proximity to visually responsive neurons and the probe replaced if necessary.

Mice were left for ~45 minutes after the initial testing for light responses, to dark adapt and to allow neuronal activity to stabilize after probe insertion. Signals (ampilified x3000) were acquired using a Recorder64 recorder system (Plexon), high-pass filtered (300 Hz), digitized at a rate of 40 kHz and then stored for offline analysis. Multichannel, multiunit recordings were analysed in Offline sorter (Plexon). Cross-channel artefacts (identical events appearing across multiple channels) were identified and removed. Principal component based sorting was used to discriminate single units, identifiable as a distinct cluster of spikes in principal component space with a clear refractory period in their interspike interval distribution. Each channel was analyzed separately, with single-unit and unclassified waveforms combined to form multiunit data for each channel. Following spike sorting, data were exported to Neuroexplorer (Nex Technologies, Littleton, MA, USA) and MATLAB R2007a (The Mathworks Inc., Natick, MA, USA) for construction of peristimulus histograms and further analysis.

### Histological analysis

**Figure 7:**
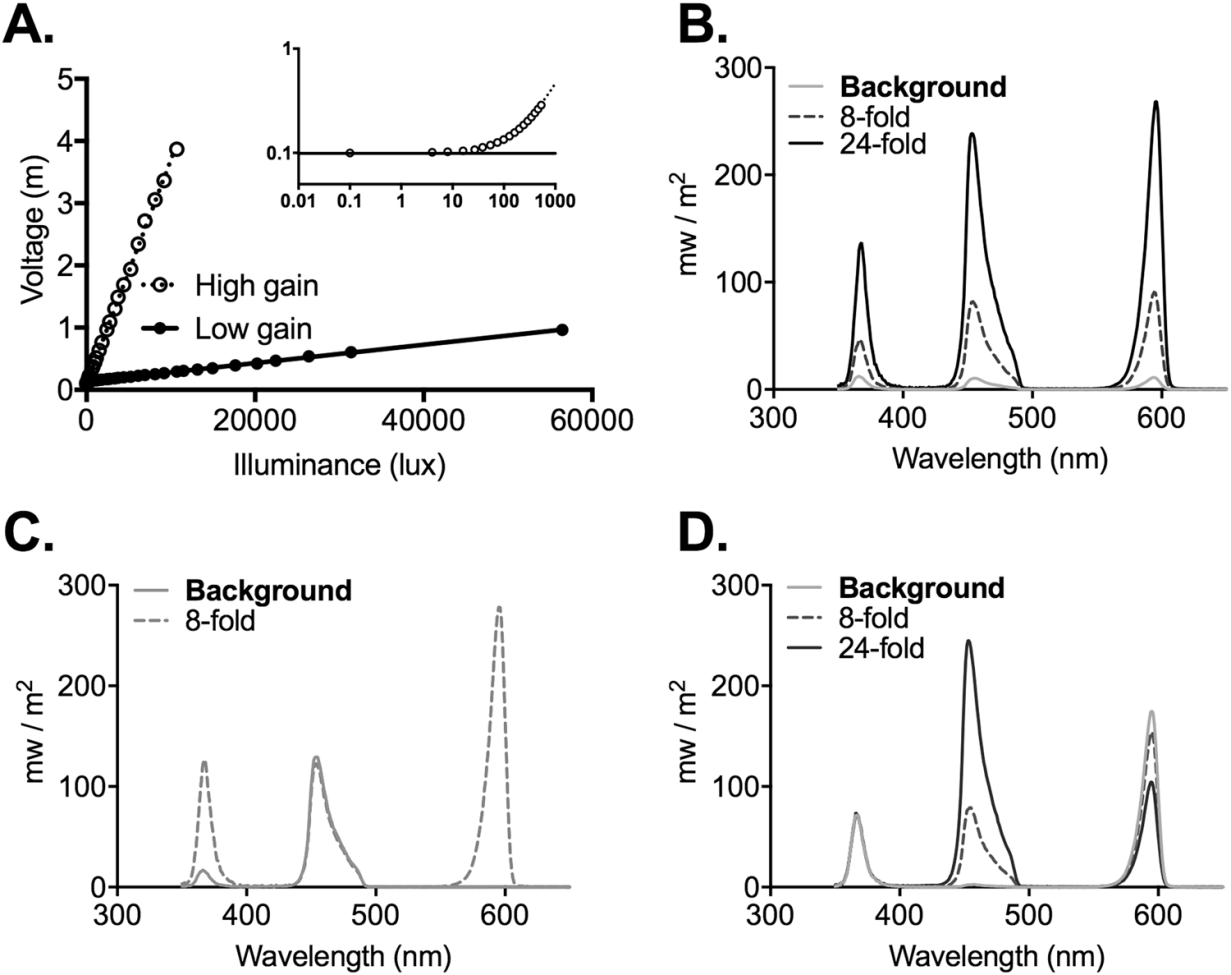
Photodiode calibration and visual stimuli. **(A)** Relationship between photodiode voltage and illuminance for the illuminance logging device at high (closed circles) and low (open circles) gain settings. Lines represent fit to linear portions of the relationship for high (solid line; mV = 0.359 lux + 105) and low (dotted line; mV = 0.0147 lux + 137) gain. Inset shows data for low gain setting across low illuminances on a log:log scale; dotted line depicts linear fit for full dataset; solid line voltage in dark. (**B**) Spectral power distribution of the 3 LED light source at the brightest background (grey) and for steps producing spectrally neutral x8 (dotted line) and x24 (black line) increases in irradiance. (**C**) Spectral power distribution of background (solid) and step (dotted line) stimuli used to produce 8x ‘cone only’ modulation. (**D**) Spectral power distribution of background (light blue) and step stimuli used to produce ‘melanopsin only’ 8x and 24x increments (dotted and dark blue lines respectively).

Electrode probe placement was verified histologically for each experiment. Before insertion into the brain for recording, the electrode was immersed in fluorescent dye (Cell Tracker CM-DiI; Invitrogen Ltd, Paisley, UK). Upon completion of the recordings the brain was removed, post fixed overnight in 4% paraformaldehye, and then cryoprotected with 30% sucrose. A sledge microtome was used to section the brain into 100 µm coronal sections, which were then mounted with vectashield (Vector Laboratories Ltd, Peterborough, UK) and coverslipped. Slides were visualized using an Olympus BX51 microscope with appropriate filter sets in order to detect the DiI fluorescence indicating the position of the electrode shanks (Fig. 7A). To estimate the anatomical location of each recording site, sections were aligned with the corresponding mouse atlas sections^30^.

### Recording Light Exposure in Human Volunteers

Data was recorded using human volunteers (postgraduate students and staff) during normal daily activities (commuting, working in an office, activity during office hours and travelling home). Volunteers wore a small silicon photodiode/accelerometer (model S1133, Hamamatsu Photonics, Japan.) attached to a modified pair of spectacles presenting a detecting surface parallel with the plane of the subject’s cornea. An intrinsic optical filter allowed the spectral response properties of the photodiode detector to approximate V(l), the photopic efficiency function, and thus to measure illuminace. As it is not feasible to record full power spectral densities under these conditions, we accept this 1 dimensional unit as being a reasonable approximation of the relative change in irradiance over time, while noting that it does not necessarily provide an accurate indication of the effective irradiance for all photoreceptors under all conditions.

The photodiode was connected to an Arduino data logging device to sample voltage at 5 Hz via a circuit that could be switched between two resistors to alter the gain of the illuminance:voltage relationship. The device was calibrated using a single white, pulse width modulated LED whose illuminance was independently measured using a spectrometer (Spectral Mk II, Cambridge Research System, UK). Separate linear functions for translating voltage into illuminance were defined for low and high gain settings (Fig. 7B) with the former being suitable for illuminances up to 7000 lux and the latter for higher values. Linearity was retained across lower illuminances (<1000 lux), although the measured voltage approached that recorded in the dark noise at very dim light (<50 lux).

We tested the sensitivity of the photodiode to variations in the incidence angle for light and found that it could be described by the generalised equation Lux = α + (b cos (cΘ)). Where α is a fitting constant and b alters the rate of illuminance decay. The fact that illuminance closely follows cos fitting curves suggests that the detector effectively detects irradiance according to Lambert’s cosine law.

### Analysis of illuminance signals

Illuminance data (n = 11 recordings of 0.6–3 h each; total:14.3 h) were analysed in Matlab. Power spectral densities were estimated from each recording using Welch’s method (Matlab function: *pwelch*) and, for presentation, combined as a weighted average as a function of recording length. Illuminance data were then filtered in forward and reverse directions to extract ‘low’, ‘mid’ and ‘high’ frequency components (6^th^ order Butterworth filters; cut on >0.032, 0.032–0.32 & <0.32 Hz respectively). Low frequency filtered data were further normalised by subtracting the mean illuminance for each recording. The distribution of illuminance values and moment-moment variations in illuminance (instaneous change between local minima and maxima) within each frequency band (across the full dataset) were then extracted for display.

### Visual stimuli

Light was measured using a calibrated spectroradiometer (Bentham Instruments, Reading, UK). Full field visual stimuli were generated using a custom-made light source (Cairn Research) consisting of individually controlled UV (λmax: 365 nm), blue (λmax: 460 nm) and red (λmax: 630 nm) LEDs. Programs written in LabVIEW (version 8; National Instruments) were used to alter stimulus intensity by controlling LED output and introducing neutral density filters using a filter wheel in the light path. Light was supplied to the subject over a 5-mm-diameter circle of opal diffusing glass (Edmund Optics Inc., York, UK) placed <1 mm from the eye contralateral to the recording site. The ipsilateral eye was illuminated with a 460 nm LED, which was maintained at a constant background level, approximately matched to the intensity of the experimental stimuli.

Simple light steps, ramps and sinusoids were produced using matched increases in the output of all three LEDs (Fig. 7C). The principles of receptor silent substitution were applied to produce steps strongly biased towards modulating effective irradiance for cones vs melanopsin and rods. Full details on estimating effective irradiance for each of the mouse photoreceptors are provided in ref. 31. In brief, this parameter was estimated by multiplying the spectral power distribution of each light setting by the *in vivo* spectral sensitivity of each photopigment class (estimated by mutliplying photopigment spectral efficiency by spectral transmission properties of the mouse lens). Using this method it was possible to use modulations in the relative output of the three LEDs to produce changes in spectral power distribution that represented large changes in effective irradiance for some photoreceptors, and minimal changes for others. In the case of ‘cone only’stimuli, large increases in UV and red LED output accompanied by small changes in blue (Fig. 7D), produced stimuli modulating SWS and LWS cone opsin effective irradiance by 2x, 4x or 8x (Michelson contrast 33%, 60% or 78% respectively) while leaving rod and melanopsin effective irradiance relatively unchanged (<4% and <9% Michelson contrast for melanopsin and rods respectively). Conversely, balanced changes in red and blue LEDs (Fig. 7E) could modulate melanopsin- and rod-effective irradiance by up to x24 (92% Michelson contrast) respectively, while minimising changes to SWS- and LWS-effective irradiance (<1% and <6% Michelson contrast for SWS and LWS respectively). As rod activity should be substantially suppressed at the bright backgrounds used here we refer to these latter stimuli as ‘melanopsin only’ for simplicity. The brightest backgrounds employed here (‘bright’) had melanopsin-effective irradiance around 5.72 × 10^13^ photons/cm^2^/s, equivalent to a mouse’s experience of daylight at low solar angles, or a bright indoor light (of around 1000 lux), 1.37 × 10^14^ photons/cm^2^/s. Neutral density filters were used to reduce intensity by 10x or 100x to produce ‘moderate’ and ‘dim’ conditions.

### Data analysis

Light responsive units were identified according to responses to 1 s stimuli as those where the peristimulus average showed a clear peak (or trough) that exceeded the 99% confidence limits estimated from a Poisson distribution derived from the prestimulus count spikes. Normalised responses were calculated by dividing the baseline subtracted firing rate of each unit to any given stimulus by the largest value for that parameter from that cell under any of the stimuli within that protocol. All data were visualised using custom-made programs in Matlab (r2008a; Mathworks), or in Office Excel (2003; Microsoft Corporation) or GraphPad (version 4.02; GraphPad Software).

## Electronic supplementary material

Supplementary Information

## References

[1] Brown TM (2016). Using light to tell the time of day: sensory coding in the mammalian circadian visual network. J Exp Biol.

[2] Berson DM, Dunn FA, Takao M (2002). Phototransduction by retinal ganglion cells that set the circadian clock. Science.

[3] Hattar S, Liao HW, Takao M, Berson DM, Yau KW (2002). Melanopsin-containing retinal ganglion cells: architecture, projections, and intrinsic photosensitivity. Science.

[4] Provencio I, Rollag MD, Castrucci AM (2002). Photoreceptive net in the mammalian retina. This mesh of cells may explain how some blind mice can still tell day from night. Nature.

[5] Baver SB, Pickard GE, Sollars PJ (2008). Two types of melanopsin retinal ganglion cell differentially innervate the hypothalamic suprachiasmatic nucleus and the olivary pretectal nucleus. Eur J Neurosci.

[6] Guler AD (2008). Melanopsin cells are the principal conduits for rod-cone input to non-image-forming vision. Nature.

[7] Belenky MA, Smeraski CA, Provencio I, Sollars PJ, Pickard GE (2003). Melanopsin retinal ganglion cells receive bipolar and amacrine cell synapses. J Comp Neurol.

[8] Dacey DM (2005). Melanopsin-expressing ganglion cells in primate retina signal colour and irradiance and project to the LGN. Nature.

[9] Lall GS (2010). Distinct contributions of rod, cone, and melanopsin photoreceptors to encoding irradiance. Neuron.

[10] Altimus CM (2010). Rod photoreceptors drive circadian photoentrainment across a wide range of light intensities. Nat Neurosci.

[11] Walmsley L (2015). Colour as a signal for entraining the mammalian circadian clock. PLoS Biol.

[12] Mouland JW, Stinchcombe AR, Forger DB, Brown TM, Lucas RJ (2017). Responses to spatial contrast in the mouse suprachiasmatic nuclei (SCN). Current Biology.

[13] Groos GA, Mason R (1980). The visual properties of rat and cat suprachiasmatic neurones. J Comp Physiol A.

[14] Aggelopoulos NC, Meissl H (2000). Responses of neurones of the rat suprachiasmatic nucleus to retinal illumination under photopic and scotopic conditions. J Physiol.

[15] Vartanian GV, Zhao X, Wong KY (2015). Using Flickering Light to Enhance Nonimage-Forming Visual Stimulation in Humans. Invest Ophthalmol Vis Sci.

[16] Brown TM, Wynne J, Piggins HD, Lucas RJ (2011). Multiple hypothalamic cell populations encoding distinct visual information. J Physiol.

[17] Meijer JH, Watanabe K, Schaap J, Albus H, Détári L (1998). Light responsiveness of the suprachiasmatic nucleus: long-term multiunit and single-unit recordings in freely moving rats. J Neurosci.

[18] Smallwood PM (2003). Genetically engineered mice with an additional class of cone photoreceptors: implications for the evolution of color vision. Proc Natl Acad Sci USA.

[19] Allen AE (2014). Melanopsin-driven light adaptation in mouse vision. Curr Biol.

[20] Drouyer E, Rieux C, Hut RA, Cooper HM (2007). Responses of suprachiasmatic nucleus neurons to light and dark adaptation: relative contributions of melanopsin and rod-cone inputs. J Neurosci.

[21] Dkhissi-Benyahya O, Gronfier C, De Vanssay W, Flamant F, Cooper HM (2007). Modeling the role of mid-wavelength cones in circadian responses to light. Neuron.

[22] al Enezi J (2011). A “Melanopic” Spectral Efficiency Function Predicts the Sensitivity of Melanopsin Photoreceptors to Polychromatic Lights. Journal of Biological Rhythms.

[23] Nelson DE, Takahashi JS (1999). Integration and saturation within the circadian photic entrainment pathway of hamsters. Am J Physiol.

[24] Zeitzer JM, Ruby NF, Fisicaro RA, Heller HC (2011). Response of the human circadian system to millisecond flashes of light. PLoS One.

[25] Zeitzer JM, Fisicaro RA, Ruby NF, Heller HC (2014). Millisecond flashes of light phase delay the human circadian clock during sleep. J Biol Rhythms.

[26] Vidal L, Morin LP (2007). Absence of normal photic integration in the circadian visual system: response to millisecond light flashes. J Neurosci.

[27] Gooley JJ (2012). Melanopsin and rod-cone photoreceptors play different roles in mediating pupillary light responses during exposure to continuous light in humans. J Neurosci.

[28] Lucas RJ, Lall GS, Allen AE, Brown TM (2012). How rod, cone, and melanopsin photoreceptors come together to enlighten the mammalian circadian clock. Prog Brain Res.

[29] Yu YQ, Barry DM, Hao Y, Liu XT, Chen ZF (2017). Molecular and neural basis of contagious itch behavior in mice. Science.

[30] Paxinos, G. F. & Franklin, K. G. J. *The mouse brain in stereotaxic coordinates: Second Edition*. (Academic Press, 2001).

[31] Brown TM (2013). The melanopic sensitivity function accounts for melanopsin-driven responses in mice under diverse lighting conditions. PLoS One.

