# Supplementary material for: The impact of temporal modulations in irradiance under light adapted conditions on the mouse suprachiasmatic nuclei (SCN)
Source: Sci Rep. 2017 Sep 5;7:10582. doi: 10.1038/s41598-017-11184-2 (PMC5585163; doi:10.1038/s41598-017-11184-2)
Supplement: Supplementary file 1 — Supplementary Information [file 41598_2017_11184_MOESM1_ESM.docx]

**The impact of temporal modulations in irradiance under light adapted conditions on the mouse suprachiasmatic nuclei (SCN)**

Rachel Dobb, Franck Martial, Daniel Elijah, Riccardo Storchi, Timothy M Brown, Robert J Lucas

**Supplementary Figure 1**

**
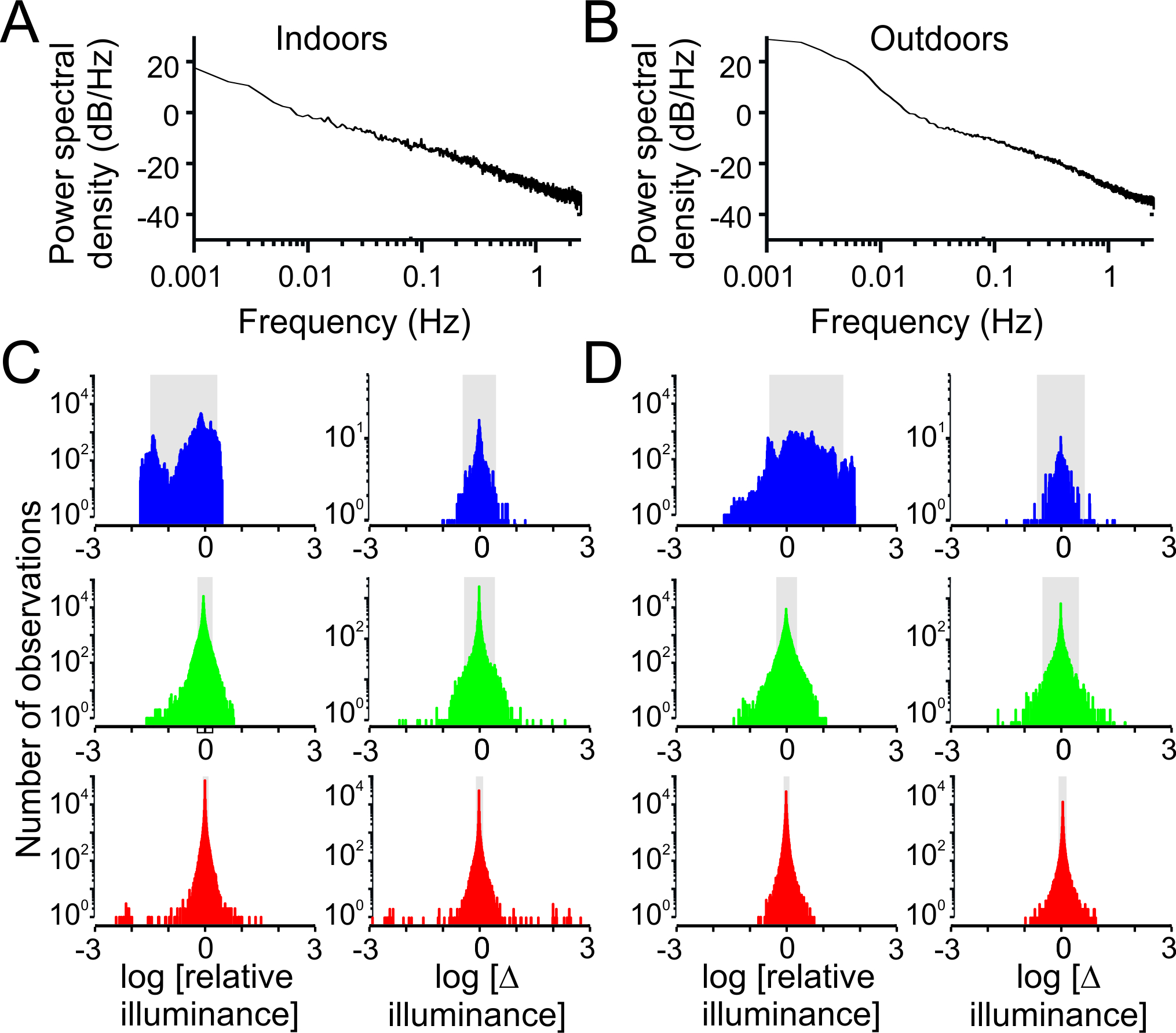
**

**Suppl. Figure 1.** Effect of environment on commonly encountered variations in illuminance. (**A**,**B**) Power spectral densities for illuminance recordings spanning epochs where the subject was entirely indoors (**A**; weighted average of 7.1h of data comprising 16 segments of length 4.1-168.8mins) or outdoors (**B**; 4h of data comprising 22 segments of length 4.4 to 45min).

(**C,D**) Histograms showing distribution of illuminance values (relative to mean for each recording epoch; left) or moment to moment changes in illuminance (delta between local minima and maxima) for indoor (**C**) or outdoor (**D**) recordings. ‘Low’, ‘mid’ and ‘high’ frequency bands extracted as in Fig 1B. Shaded regions represent the central 95% of each data distribution. Overall mean illuminance for epochs contributing to **A**&**C** and **B**&**D** was 2.16±0.31 and 3.87±0.36 log lux respectively (mean±SD).
